# Alcohol, Drugs and Addictive Behaviors in ICD-11: major changes have improved the diagnostic system

**DOI:** 10.1192/j.eurpsy.2025.76

**Published:** 2025-08-26

**Authors:** J. B. Saunders

**Affiliations:** National Centre for Youth Substance Use Research, The University of Queensland, Brisbane, Queensland 4072, Australia

## Abstract

**Abstract:**

The latest revision of the International Classification of Diseases, ICD-11, was first issued by the World Health Organization in 2022 and a comprehensive account of mental and behavioural disorders, the Clinical Descriptions and Diagnostic Requirements (CDDR) was published in 2024. Within mental disorders is a new section “Disorders due to Substance Use and Addictive Behaviours”, which sets out the diagnostic requirements for, and distinctions between, the accepted range of conditions related to psychoactive substance use and behaviours such as gambling and online (video-) gaming.

Considerable changes have been made to ICD-11 compared with its predecessor ICD-10, originally published in 1992. The process leading to ICD-11 began in 2005 and a work group for substance disorders was convened in 2010, and for addictive behaviours in 2014. Certain key considerations guided the development of the new section, which had to serve both public health and preventive and clinical diagnostic needs. These were:

1. the importance for reporting a wide range of psychoactive substances for monitoring and prevention, which led to the expansion to 14 categories;
2. the need on public health and also clinical grounds for having a spectrum of severity of substance use, which could be defined precisely in line with the evidence base;
3. the recognition of the high-level psychometric performance of key diagnoses in ICD-10 including substance dependence; and:
4. compatibility with the available data on the neurobiology and cognitive processes underlying addictive disorders.

Four central substance use diagnoses feature in ICD-11. They are, in order of severity, (i) hazardous substance use, (ii) episode of harmful use, (iii) harmful (pattern of) substance use, and (iv) substance dependence. When there is any form of unhealthy use, one of these four conditions should be diagnosed. In addition, there are many substance-induced disorders, including intoxication, withdrawal, substance-induced delirium, substance-induced mental disorders, and substance-induced neurocognitive disorders. In separate sections are the physical disorders related to substance use. There are currently two diagnoses available for specific behavioural conditions, namely gambling disorder and gaming disorder, together with the health risk factors of hazardous gambling and hazardous gaming. The presentation will include some of the empirical data that support the election of these diagnoses which form the basis for monitoring and healthcare provision in WHO (UN) member countries.

**Disclosure of Interest:**

None Declared

